# Noninvasive Detection of Clinically Significant Fibrosis in Biopsy-Proven MASLD: A Retrospective Cross-Sectional Diagnostic Accuracy Study

**DOI:** 10.3390/medicina62081527

**Published:** 2026-08-08

**Authors:** Onur Eksi, Kadri Atay, Tugce Eskazan, Elgun Abishov, Mustafa Canbakan, Billur Canbakan

**Affiliations:** 1 Department of Endocrinology and Metabolism, Faculty of Medicine, Istanbul Medipol University, Istanbul 34214, Turkey; 2 Department of Gastroenterology, Mardin Training and Research Hospital, Mardin 47100, Turkey; dr_kadrii@yahoo.com; 3 Division of Gastroenterology, Department of Internal Medicine, Cerrahpaşa Faculty of Medicine, Istanbul University-Cerrahpaşa, Istanbul 34098, Turkey; drtugcecaliskan@gmail.com (T.E.); billur.canbakan@iuc.edu.tr (B.C.); 4 Department of Internal Medicine, Hisar Intercontinental Hospital, Istanbul 34768, Turkey; elgunabishovctf@gmail.com; 5 Department of Nephrology, School of Medicine, Haydarpaşa Numune Training and Research Hospital, University of Health Sciences, Istanbul 34000, Turkey; dr.mcanbakan@gmail.com

**Keywords:** metabolic dysfunction-associated steatotic liver disease, liver fibrosis, transient elastography, biomarkers, sensitivity and specificity

## Abstract

*Background and Objectives*: Metabolic dysfunction-associated steatotic liver disease (MASLD) is associated with substantial morbidity, and hepatic fibrosis stage is the strongest predictor of long-term outcomes. Although liver biopsy remains the reference standard for fibrosis assessment, its invasiveness has driven the development of noninvasive diagnostic tools, including transient elastography (TE) and serum-based fibrosis scores. To evaluate and compare the diagnostic performance of TE and commonly used serum-based fibrosis scores for detecting clinically significant fibrosis (≥F2) in patients with biopsy-proven MASLD. *Materials and Methods*: This retrospective cross-sectional exploratory diagnostic accuracy study included 30 adults with biopsy-confirmed MASLD. Patients were classified into clinically significant fibrosis (CSF, ≥F2; *n* = 9) and no/mild fibrosis (NMF, F0–F1; *n* = 21) groups. Among the nine patients with CSF, six had stage F2 fibrosis and three had stage F3 fibrosis. TE, FIB-4, APRI, NAFLD Fibrosis Score (NFS), BARD score, and HOMA-IR were evaluated. Diagnostic performance was assessed using receiver operating characteristic (ROC) analysis, optimal cutoff values, dual-cutoff strategies, and confusion matrix analysis. *Results*: TE, FIB-4, and APRI demonstrated the highest diagnostic performance (AUROC: 0.93 for each). TE showed the most balanced diagnostic accuracy (sensitivity 88.9%, specificity 90.5%), whereas APRI provided the highest specificity (95.2%) and the strongest positive likelihood ratio (+LR: 18.67). FIB-4 achieved 100% sensitivity with lower specificity (76.2%). NFS demonstrated moderate diagnostic performance (AUROC: 0.83) with a relatively wide gray zone (56.7%), whereas BARD and HOMA-IR showed limited discriminative ability. When fibrosis categories automatically assigned by the TE device were evaluated, the overall diagnostic accuracy was 93.3%. *Conclusions*: TE, FIB-4, and APRI demonstrated promising diagnostic performance as noninvasive tools for identifying clinically significant fibrosis in biopsy-proven MASLD. These findings generate the hypothesis that a sequential diagnostic strategy using serum-based fibrosis scores for initial risk stratification followed by TE for confirmation may reduce the need for liver biopsy. However, this hypothesis requires confirmation in larger prospective validation studies.

## 1. Introduction

Cirrhosis and hepatocellular carcinoma remain the most serious complications of metabolic dysfunction-associated steatotic liver disease (MASLD), formerly known as nonalcoholic fatty liver disease (NAFLD), and account for an increasing proportion of liver-related morbidity and mortality worldwide [1,2,3,4]. Hepatic fibrosis is the strongest histological predictor of liver-related outcomes, overall mortality, and progression to cirrhosis. Although advanced fibrosis (stages F3–F4) has traditionally been regarded as the principal prognostic threshold, increasing evidence indicates that clinically significant fibrosis (≥F2) represents the earliest stage at which disease progression accelerates and therapeutic intervention becomes clinically meaningful [5,6].

Current international practice guidelines recommend the identification of patients with clinically significant fibrosis because they require closer clinical surveillance, specialist hepatology assessment, and consideration of disease-modifying therapy when appropriate [6,7]. The recent approval of resmetirom, the first pharmacological therapy specifically indicated for patients with non-cirrhotic MASH and moderate-to-advanced fibrosis, has further increased the clinical importance of accurately identifying patients with fibrosis stage ≥ F2 [5]. Consequently, reliable noninvasive detection of clinically significant fibrosis has become a major objective in both routine clinical practice and clinical research.

Although liver biopsy remains the reference standard for fibrosis staging, its invasive nature, sampling variability, interobserver variability, procedure-related complications, and limited feasibility for repeated assessment substantially restrict its routine clinical use [8]. Consequently, several noninvasive methods have been developed to estimate hepatic fibrosis, including serum-based biomarkers and imaging techniques such as transient elastography (TE). Among these, the Fibrosis-4 (FIB-4) index is widely recommended as the first-line screening tool because of its simplicity, accessibility, and cost-effectiveness [6]. Other widely used serum-based fibrosis scores include the NAFLD Fibrosis Score (NFS), the aspartate aminotransferase-to-platelet ratio index (APRI), and the BARD score, all of which have demonstrated varying diagnostic performance across different patient populations [1,8,9,10,11].

Despite their widespread clinical use, the comparative diagnostic accuracy of these noninvasive tests for identifying clinically significant fibrosis (≥F2) remains insufficiently characterized, particularly in biopsy-proven MASLD cohorts. Furthermore, evidence directly comparing transient elastography with commonly used serum fibrosis scores using liver biopsy as the reference standard remains limited. Therefore, the present exploratory diagnostic accuracy study aimed to evaluate and compare the diagnostic performance of transient elastography, FIB-4, NFS, APRI, and the BARD score for detecting clinically significant fibrosis (≥F2) in patients with biopsy-proven MASLD, with liver biopsy serving as the reference standard. The findings are intended to provide preliminary evidence to inform future prospective validation studies.

## 2. Materials and Methods

### 2.1. Study Design and Setting

We conducted a retrospective, cross-sectional diagnostic accuracy study among adults with biopsy-confirmed metabolic dysfunction-associated steatotic liver disease (MASLD) attending the Gastroenterology and Hepatology Outpatient Clinic, Faculty of Medicine, İstanbul University–Cerrahpaşa. The study was approved by the local ethics committee (Approval No. 121098; 7 August 2019) and was conducted in accordance with the principles of the Declaration of Helsinki. The requirement for written informed consent was waived because of the retrospective design of the study and the anonymization of patient data.

### 2.2. Participants

**Figure 1:**
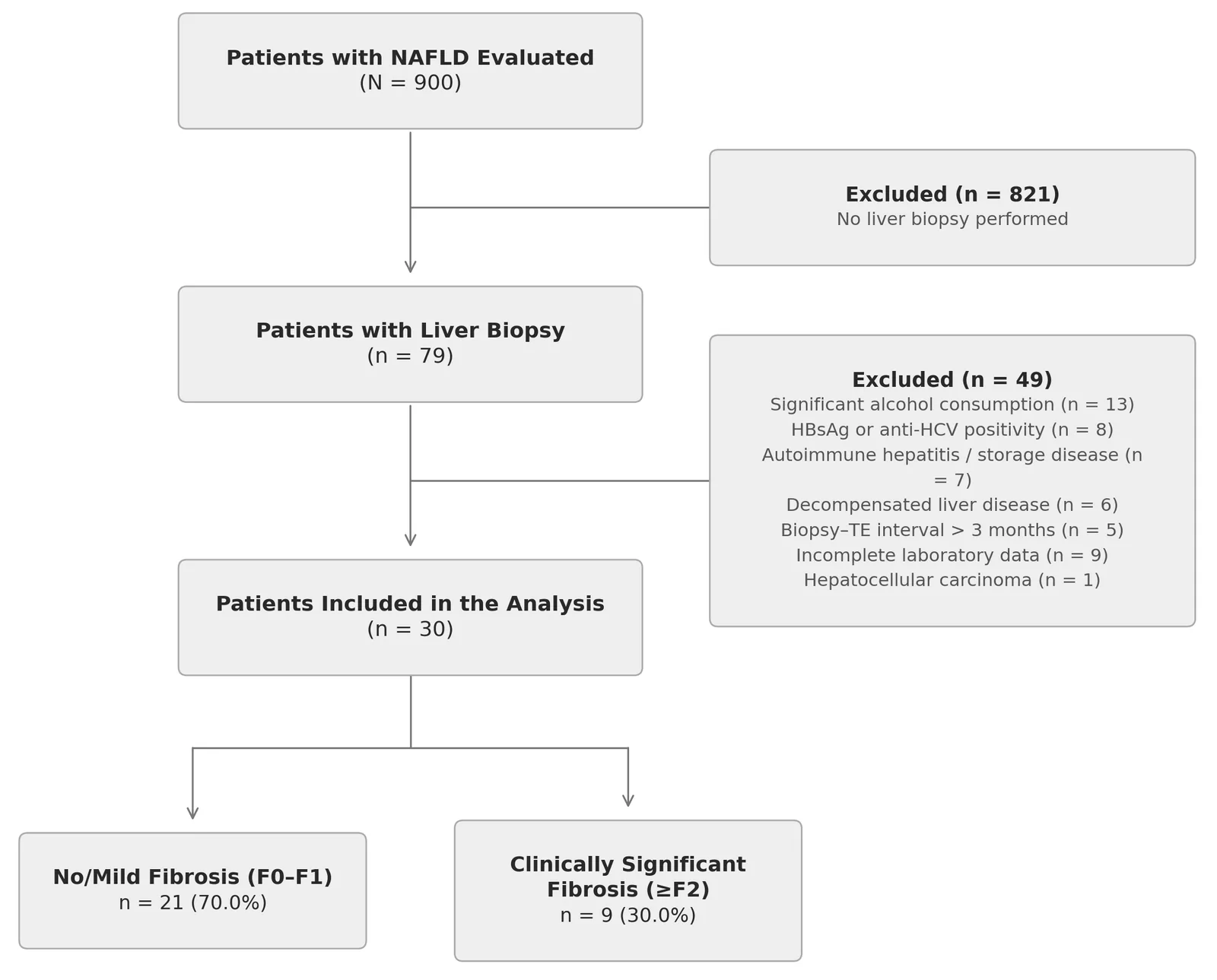
Patient selection flowchart (STARD 2015) [12].

Medical records of patients who presented between January 2015 and December 2019 were reviewed. Patients aged 18 years or older with biopsy-proven MASLD and no identifiable secondary cause of hepatic steatosis were eligible for inclusion. Patients were excluded if they had any of the following: significant alcohol consumption (≥30 g/day for men and ≥20 g/day for women); hepatitis B surface antigen (HBsAg) positivity; anti-hepatitis C virus (anti-HCV) antibody positivity; autoimmune hepatitis; or any other chronic liver disease, including Wilson’s disease and hereditary hemochromatosis. All participants underwent routine HIV serological testing, and all were HIV seronegative. After applying these eligibility criteria, 30 patients with biopsy-proven MASLD were included in the final analysis (Figure 1).

### 2.3. Data Collection

Patient records were reviewed, and sociodemographic, morphometric, and laboratory data were extracted for all participants, together with findings from ultrasonography, transient elastography (FibroScan), and liver biopsy. The upper limits of normal were defined according to the laboratory reference ranges as follows: AST > 40 U/L, ALT > 33 U/L, and gamma-glutamyl transferase (GGT) > 45 U/L.

### 2.4. Definitions and Cutoff Values

Body mass index (BMI) was calculated as body weight (kg) divided by height squared (m^2^). Patients with a BMI ≥ 30 kg/m^2^ were classified as obese [13]. Elevated waist circumference was defined as >90 cm in women and >100 cm in men [14].

The homeostasis model assessment of insulin resistance (HOMA-IR) was used to assess insulin resistance (IR) and was calculated using the following formula: HOMA-IR = [fasting insulin (μIU/mL)] × [fasting glucose (mg/dL)]/405. A HOMA-IR value ≥ 2.5 was considered indicative of insulin resistance [15].

The Fibrosis-4 (FIB-4) index, NAFLD Fibrosis Score (NFS), aspartate aminotransferase-to-platelet ratio index (APRI), and BARD score were calculated for each participant [16,17]. The BARD score is based on BMI, the AST/ALT ratio, and the presence of newly diagnosed or pre-existing type 2 diabetes mellitus. The NFS incorporates age, hyperglycemia, BMI, platelet count, albumin level, and the AST/ALT ratio. The FIB-4 index incorporates age, platelet count, and ALT and AST levels [17].

For the identification of clinically significant fibrosis, the following cutoff values were applied: FIB-4 ≥ 1.3, NFS ≥ −0.676, BARD ≥ 2, and APRI ≥ 1.0 [3,16,18].

### 2.5. Assessment of Hepatic Steatosis and Fibrosis

Hepatic steatosis and fibrosis were assessed using transient elastography (FibroScan^®^; Echosens, Paris, France). The FibroScan^®^ device measures liver fat content using the controlled attenuation parameter (CAP), expressed in decibels per meter (dB/m), and liver stiffness, expressed as Young’s modulus in kilopascals (kPa) [19].

All liver biopsy specimens were evaluated by a single expert hepatopathologist using the Brunt classification system for histopathological staging [20,21]. Steatosis was graded on a scale of 0 to 3, lobular inflammation on a scale of 0 to 3, and fibrosis on a scale of 0 to 4. Clinically significant fibrosis was defined as stage F2 or higher (≥F2), advanced fibrosis as stage F3 or higher (≥F3), and cirrhosis as stage F4 [7,16].

Patients with histologically confirmed clinically significant fibrosis (≥F2) were assigned to the clinically significant fibrosis (CSF) group, whereas those with no fibrosis or mild fibrosis (F0–F1) were assigned to the non-clinically significant fibrosis (NMF) group.

### 2.6. Statistical Analysis

The normality of continuous variables was assessed using the Shapiro–Wilk test. However, given the limited statistical power of this test in a small sample (*n* = 30; 21 vs. 9) and the associated risk of Type II error, a nonparametric approach was adopted for all continuous variables. Comparisons between the clinically significant fibrosis (≥F2) and mild fibrosis (F0–F1) groups were performed using the Mann–Whitney U test for continuous variables and Fisher’s exact test for categorical variables because the expected cell frequencies were less than five. Continuous variables were expressed as median [minimum–maximum], and categorical variables as *n* (%). The BARD score, an ordinal variable, was also reported as median [minimum–maximum]. Effect sizes were calculated as r = |Z|/√N for nonparametric comparisons and as exact conditional odds ratios with 95% confidence intervals for categorical comparisons. The robustness of statistically significant findings was evaluated using complete enumeration of all possible allocations (exact permutation test).

For the diagnostic performance analysis, the discriminative ability of five noninvasive tests (TE liver stiffness, FIB-4, NFS, APRI, and BARD) for identifying clinically significant fibrosis was evaluated using empirical (nonparametric) receiver operating characteristic (ROC) analysis. Because only nine patients had the target condition, the asymptotic normality assumption underlying the DeLong method was considered unreliable; therefore, the area under the ROC curve (AUROC) confidence intervals were estimated using the percentile bootstrap method with 2000 iterations. Optimal cutoff values were determined using the Youden index (J = sensitivity + specificity − 1). The 95% confidence intervals for sensitivity and specificity were calculated using the Clopper–Pearson exact method, whereas those for positive and negative likelihood ratios were calculated using the Simel method. Positive and negative predictive values were calculated based on the observed sample prevalence (30.0%). Because the TE categorical classification was based on the device-generated fibrosis stage threshold (≥F3), AUROC analysis was not performed for this categorical classification; only sensitivity, specificity, and classification performance metrics were reported.

Dual cutoff thresholds recommended by the EASL 2021 and AASLD 2023 clinical practice guidelines were applied to stratify patients into low-risk, gray-zone, and high-risk categories (FIB-4: <1.30/>2.67; NFS: <−1.455/>0.676; APRI: <0.50/>1.00) [22,23]. The original cutoff of ≥ 2 was applied for the BARD score, whereas the device-generated fibrosis stage threshold (≥F3) was used for the TE categorical classification. For each test, the proportion of patients with clinically significant fibrosis within each risk category, the number of missed clinically significant fibrosis cases, and the width of the gray zone were reported. Confusion matrix analyses were based on the Youden-derived optimal cutoffs obtained from the ROC analysis. For HOMA-IR, the predefined clinical cutoff of ≥2.5 was used, whereas the device-generated fibrosis stage threshold (≥F3) was applied for the TE categorical classification.

A two-sided *p*-value ≤ 0.05 was considered statistically significant throughout the analyses. Statistical analyses were performed using Jamovi (version 2.6.44, The Jamovi Project, 2023, https://www.jamovi.org) and JASP (version 0.19.3, Jeffreys’ Amazing Statistics Program, 2024, https://jasp-stats.org).

## 3. Results

Clinically significant fibrosis (Brunt stage ≥ F2) was identified in 9 patients (30.0%), including 6 patients with stage F2 and 3 patients with stage F3 (CSF group), while 21 patients (70.0%) had no or mild fibrosis (F0–F1) (NMF group).

The two groups were well matched with respect to age, sex, BMI, and waist circumference (all *p* > 0.05; Table 1).

Among the liver enzymes, only AST levels were significantly higher in the CSF group than in the NMF group (*p* = 0.048; r = 0.36). The proportion of patients with elevated AST (>40 U/L) was markedly higher in the CSF group than in the NMF group (*p* = 0.019; OR = 14.15, 95% CI: 1.11–825.63) (Table 2). Platelet counts were significantly lower in the CSF group than in the NMF group (*p* < 0.001; r = 0.67).

TE liver stiffness values were significantly higher in the CSF group than in the NMF group (*p* < 0.001; r = 0.67). The proportion of patients with advanced fibrosis (≥F3) according to the device-generated TE classification was also significantly higher in the CSF group (*p* < 0.001). Among the noninvasive fibrosis scores, FIB-4 (*p* < 0.001; r = 0.67), APRI (*p* < 0.001; r = 0.67), and NFS (*p* = 0.006; r = 0.51) were all significantly higher in the CSF group. Likewise, the proportions of patients exceeding the established cutoffs were significantly greater in the CSF group for FIB-4 (*p* = 0.002), NFS (*p* = 0.014), and APRI (*p* < 0.001). In contrast, neither the median BARD score nor the proportion of patients with BARD ≥2 differed significantly between the groups (*p* = 0.434 and *p* = 0.141, respectively). Histopathological steatosis grade (*p* = 0.370) and lobular inflammation severity (*p* = 0.195) were comparable between the groups.

**Figure 2:**
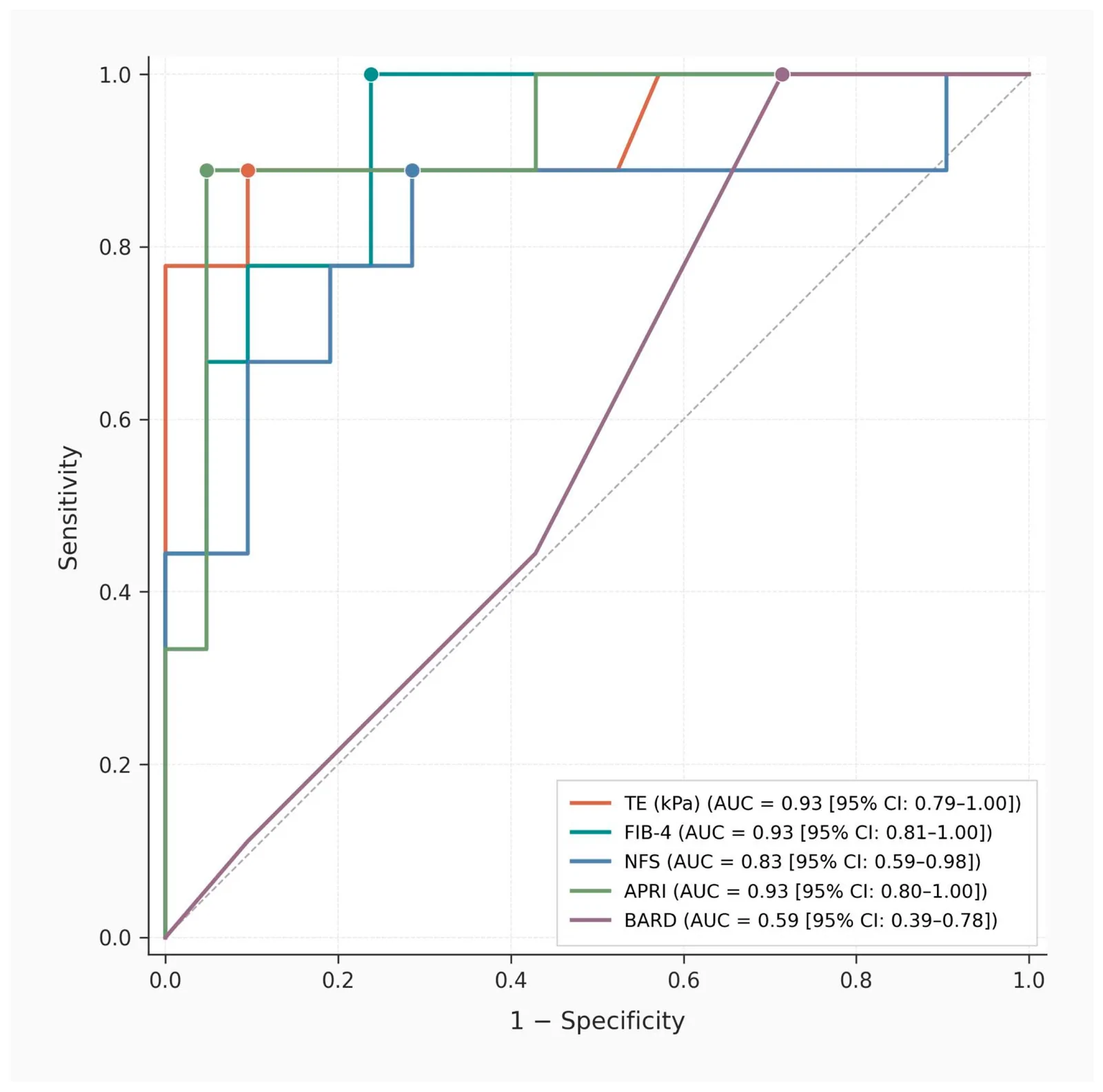
Receiver operating characteristic curves of non-invasive tests for detecting clinically significant fibrosis (≥F2) against liver biopsy. Areas under the curve with 95% confidence intervals were estimated using 2000 bootstrap resamples (percentile method). Filled circles indicate optimal cutoff points determined by the Youden index. The dashed diagonal line represents the reference line (AUC = 0.50). TE, transient elastography; FIB-4, Fibrosis-4 index; NFS, NAFLD Fibrosis Score; APRI, AST-to-Platelet Ratio Index; BARD, BARD score.

ROC analysis demonstrated excellent diagnostic performance for TE liver stiffness, FIB-4, and APRI, each achieving an AUROC of 0.93. NFS showed moderate discriminative ability (AUROC: 0.83; 95% CI: 0.59–0.98), whereas BARD demonstrated the lowest diagnostic performance (AUROC: 0.59; 95% CI: 0.39–0.78) (Table 3; Figure 2). Detailed bootstrap-derived estimates of diagnostic performance, including confidence intervals and additional performance metrics, are provided in Supplementary Table S1.

At the optimal cutoff of ≥10.70 kPa, TE liver stiffness showed the most balanced diagnostic performance, with a sensitivity of 88.9% and a specificity of 90.5%. FIB-4 (≥1.28) achieved 100% sensitivity, although its specificity was limited to 76.2%. APRI (≥0.48) provided the highest specificity (95.2%) while maintaining a sensitivity of 88.9%. In terms of positive likelihood ratios, APRI (+LR: 18.67) and TE (+LR: 9.33) demonstrated the strongest diagnostic performance. Although BARD achieved 100% sensitivity, its specificity was only 28.6%, resulting in limited clinical utility (+LR: 1.40).

The device-generated TE fibrosis stage classification (≥F3) yielded the same sensitivity (88.9%), specificity (95.2%), and positive likelihood ratio (18.67) as APRI.

In the dual-cutoff analysis, the narrowest gray zone was observed for APRI (20.0%; 6/30 patients), whereas NFS had the widest gray zone (56.7%; 17/30 patients). Both FIB-4 and NFS misclassified one patient with clinically significant fibrosis into the low-risk category (missed rate: 11.1% each). APRI misclassified two patients with clinically significant fibrosis into the low-risk category (22.2%). BARD did not miss any patient with clinically significant fibrosis; however, it classified 80.0% of patients (24/30) as high risk, substantially limiting its clinical utility. The TE categorical classification missed one patient with clinically significant fibrosis (11.1%) and produced no gray zone (Table 4).

In the confusion matrix analysis, the highest overall accuracy was observed for the TE categorical classification (93.3%), followed by TE liver stiffness and APRI (both 90.0%). FIB-4 achieved an accuracy of 83.3%, whereas NFS achieved 76.7%. BARD (50.0%) and HOMA-IR (36.7%) showed the lowest accuracy. Using the predefined clinical cutoff of ≥2.5, HOMA-IR misclassified 17 of 21 patients without clinically significant fibrosis as false positives, yielding a specificity of only 19.0%. In this analysis, both BARD and FIB-4 maintained 100% sensitivity for detecting clinically significant fibrosis; however, BARD generated 15 false-positive classifications, whereas FIB-4 generated five (Table 5).

## 4. Discussion

Among the noninvasive tools evaluated in this biopsy-confirmed MASLD cohort, TE, FIB-4, and APRI demonstrated the highest diagnostic performance for identifying clinically significant fibrosis. Among these, TE provided the most balanced combination of sensitivity and specificity, while APRI yielded the highest rule-in value with a strong positive likelihood ratio. FIB-4 achieved excellent sensitivity but at the expense of lower specificity, supporting its role as a screening rather than a confirmatory tool. NFS, by comparison, demonstrated only moderate discriminative ability, whereas BARD—despite its high sensitivity at the optimal ROC-derived cutoff—lacked sufficient specificity for meaningful clinical stratification. Collectively, these findings support the complementary roles of elastography and simple serum-based indices in the noninvasive assessment of liver fibrosis.

The equivalent AUROC of 0.93 observed for TE, FIB-4, and APRI in this cohort warrants careful interpretation. In the largest pooled analysis to date (*n* = 5735), Mózes et al. [24] reported AUROCs of 0.85 for TE, 0.76 for FIB-4, and 0.73 for NFS for advanced fibrosis (≥F3). Similarly, in a meta-analysis of 13,046 patients with NAFLD, Xiao et al. [25] reported AUROCs of 0.88 for FibroScan M-probe, 0.84 for FIB-4, 0.84 for NFS, 0.77 for APRI, and 0.76 for BARD. The convergence of TE, FIB-4, and APRI at the same AUROC in our study likely reflects the small sample size, the inclusion of only nine patients with clinically significant fibrosis, and the relatively high prevalence of clinically significant fibrosis (30%), all of which may have inflated the discriminative performance of serum-based indices. Furthermore, our use of a ≥F2 endpoint, rather than the ≥F3 threshold evaluated in most previous studies, may also have contributed to these findings. Nevertheless, the consistently strong performance of TE across both our cohort and previous studies [24,25,26] reinforces its established role in the noninvasive assessment of liver fibrosis. Notably, the empirically derived optimal TE cutoff (10.70 kPa) was close to the device-generated threshold used for advanced fibrosis (≥F3). However, this proximity should not be interpreted as validation of the device-generated threshold and most likely reflects the limited number of patients with clinically significant fibrosis in the present cohort. Furthermore, a post hoc analysis restricted to patients with histologically confirmed stage F2 fibrosis demonstrated similar overall findings, although these results should be interpreted cautiously because of the very small sample size (Supplementary Table S2).

That FIB-4 and APRI performed comparably to TE in this tertiary-center cohort likely reflects the marked thrombocytopenia and elevated AST levels observed among patients with clinically significant fibrosis, the very variables incorporated into both indices. The pathophysiological basis for these associations is well established: progressive portal hypertension and reduced thrombopoietin synthesis contribute to declining platelet counts, while ongoing hepatocellular injury increases AST concentrations [17,24,26]. When these abnormalities become sufficiently pronounced, serum-based fibrosis scores may approach the diagnostic performance of elastography.

BARD, on the other hand, yielded the weakest discrimination of all evaluated indices. Although BARD achieved 100% sensitivity at the optimal ROC-derived cutoff, its very low specificity resulted in limited clinical utility. This finding is consistent with previous studies across different populations. Xiao et al. [25] reported the lowest summary AUROC for BARD (0.76) among all serum-based indices in their meta-analysis. Shah et al. [27] demonstrated that BARD (AUROC: 0.70) was significantly inferior to FIB-4 (AUROC: 0.80) in 541 participants from the NASH Clinical Research Network. Sun et al. [28] confirmed this hierarchy in a meta-analysis of 1038 patients (AUROC: 0.76 (BARD) vs. 0.85 (FIB-4)). Ruffillo et al. [29] similarly reported an AUROC of 0.67 for BARD, with a positive predictive value of only 45.2% despite an acceptable negative predictive value. The poor specificity observed in our cohort likely reflects the metabolic homogeneity of the study population. More than half of the patients were obese, approximately two-fifths had diabetes mellitus, and nearly four-fifths had insulin resistance. Consequently, the principal BARD components—BMI, AST/ALT ratio, and diabetes status—showed limited variability between fibrosis groups, thereby reducing the discriminatory capacity of the score.

Although NFS demonstrated moderate discriminative ability, the dual-cutoff framework left 56.7% of patients (17/30) within the indeterminate gray zone, limiting its practical clinical utility in this cohort. A similarly broad gray zone (55%) was reported by Pennisi et al. [30] in an individual patient data meta-analysis of 1780 patients with diabetes and NAFLD. Mózes et al. [24] likewise demonstrated that NFS performed worse than both TE and FIB-4 (AUROC: 0.73 vs. 0.85 and 0.76, respectively). Although some studies have reported better performance for NFS [3], the broad gray zone observed in our study likely reflects the relatively limited variability in age and BMI, two major components of the NFS equation.

The additional diagnostic value of metabolic or insulin resistance markers beyond established fibrosis scores remains uncertain. Ullman et al. [1] found no improvement in diagnostic performance when CRP, HbA1c, HOMA-IR, or uric acid were added to FIB-4, whereas Arun Kumar et al. [31] reported significant correlations between HbA1c, fasting and postprandial glucose concentrations, and shear wave elastography measurements. Anthropometric measures, including BMI and waist circumference, have also been associated with advanced fibrosis in patients with type 2 diabetes mellitus [32]. Nevertheless, optimal combinations of noninvasive tests may differ across populations [11], and liver biopsy therefore remains the reference standard for fibrosis staging.

HOMA-IR did not differ significantly between fibrosis groups, and confusion matrix analysis ranked it as the poorest-performing marker in our cohort. This finding contrasts with the multicenter Japanese study by Fujii et al. [33], in which HOMA-IR independently predicted advanced fibrosis among 361 nondiabetic patients with biopsy-confirmed NAFLD. Other studies have similarly demonstrated a strong association between insulin resistance and NAFLD [15,34]. Several factors may explain this discrepancy. Nearly four-fifths of our patients had HOMA-IR values ≥ 2.5, creating a ceiling effect. Furthermore, 40% of patients had diabetes mellitus, unlike the exclusively nondiabetic population studied by Fujii et al. [33], resulting in a more homogeneous metabolic profile. In this setting, HOMA-IR lost much of its discriminatory capacity.

Current clinical practice guidelines endorse a sequential approach in which serum-based fibrosis scores are used for initial risk stratification and elastography is reserved for confirmatory assessment [24,26,35,36]. Although the present study did not evaluate a sequential diagnostic pathway, the findings are compatible with this strategy. In our dual-cutoff analysis, APRI demonstrated the narrowest gray zone, whereas NFS had the widest. The TE categorical classification missed one patient with clinically significant fibrosis but produced no gray zone, yielding the highest overall accuracy in the confusion matrix analysis. These observations are in agreement with those reported by Lee et al. [36], who demonstrated that sequential assessment using FIB-4 followed by liver stiffness measurement achieved 81% diagnostic accuracy while minimizing indeterminate results. Taken together, our findings generate the hypothesis that a sequential approach may be clinically useful; however, this hypothesis requires confirmation in larger prospective studies.

The strengths of this study include the use of liver biopsy as the reference standard and histopathological staging for all participants. Nevertheless, several limitations should be acknowledged. The retrospective design precludes causal inference, although the temporal relationship between the index tests and the reference standard was clearly established. The small sample size, particularly the inclusion of only nine patients with clinically significant fibrosis, resulted in wide confidence intervals and limited statistical power; therefore, the findings should be regarded as hypothesis-generating. The single-center tertiary referral setting and the relatively high prevalence of clinically significant fibrosis (30%) may also limit the generalizability of the results to primary care settings or populations with different disease prevalence. Although laboratory testing and transient elastography were performed using standardized protocols, retrospective extraction of pathology data from narrative reports may have introduced information bias. Multivariable analyses were not performed because the primary objective was diagnostic accuracy rather than prediction modeling, and residual confounding cannot be excluded. Complete-case analysis was used, and the potential impact of missing data was not formally assessed. No formal adjustment for multiple comparisons was performed, although permutation testing was used to evaluate the robustness of statistically significant findings. The study population was identified retrospectively from patients evaluated between 2015 and 2019. Although the current manuscript adopts the contemporary MASLD nomenclature, the original clinical records predated this terminology. Given the substantial overlap between the NAFLD and MASLD definitions, this difference is unlikely to have materially influenced the study findings [37].

In conclusion, TE, FIB-4, and APRI demonstrated promising diagnostic performance for identifying clinically significant fibrosis in this biopsy-confirmed MASLD cohort. TE provided the most balanced diagnostic profile, APRI showed the strongest rule-in capability, and FIB-4 offered excellent sensitivity, supporting its role as a first-line screening tool. In contrast, NFS showed only moderate utility, whereas BARD and HOMA-IR provided limited clinical discrimination in this metabolically homogeneous population.

These findings support the potential value of a sequential noninvasive assessment strategy combining serum-based fibrosis scores with elastography. However, given the retrospective single-center design and the limited number of patients with clinically significant fibrosis, these findings should be regarded as exploratory and hypothesis-generating until validated in larger prospective multicenter studies.

## 5. Conclusions

To summarize, TE, FIB-4, and APRI each demonstrated high diagnostic accuracy for identifying clinically significant fibrosis in this cohort of patients with biopsy-confirmed MASLD. TE provided the most balanced diagnostic performance, APRI showed the strongest rule-in value, and FIB-4 offered excellent sensitivity, supporting its role as a first-line screening tool. In contrast, NFS demonstrated moderate utility, whereas BARD and HOMA-IR were of limited clinical value in this metabolically homogeneous population.

A stepped noninvasive strategy using serum-based fibrosis scores for initial risk stratification and elastography for confirmatory assessment appears promising based on these findings and may reduce the need for liver biopsy in appropriately selected patients. However, given the retrospective single-center design and the limited number of patients with clinically significant fibrosis, these findings should be considered exploratory and hypothesis-generating and require validation in larger prospective multicenter studies.

## Figures and Tables

**Table 1:** Demographic and Anthropometric Characteristics and Comorbidities of the Study Groups.

Variable	NMF Group (*n* = 21)	CSF Group (*n* = 9)	*p*	Effect Size [95% CI]
***Demographic***				
Age (years) ^†^	59.0 [42.0–70.0]	54.0 [29.0–71.0]	0.230	r = 0.22 [−0.01–0.58]
Sex (Male), *n* (%) ^‡^	8 (38.1)	2 (22.2)	0.675	OR = 2.15 [0.36–13.05]
***Anthropometric***				
BMI (kg/m^2^) ^†^	30.0 [24.0–40.0]	30.0 [20.0–40.0]	0.570	r = 0.11 [0.01–0.48]
Waist circumference (cm) ^†^	102.0 [80.0–124.0]	98.0 [76.0–120.0]	0.140	r = 0.27 [0.02–0.59]
***Comorbidities***				
Obesity (BMI ≥ 30), *n* (%) ^‡^	12 (57.1)	5 (55.6)	1.000	OR = 1.07 [0.22–5.14]
Elevated waist circumference, *n* (%) ^‡^	19 (90.5)	6 (66.7)	0.143	OR = 4.75 [0.64–35.48]
Diabetes mellitus, *n* (%) ^‡^	8 (38.1)	4 (44.4)	1.000	OR = 0.77 [0.16–3.74]
Insulin resistance (HOMA-IR ≥ 2.5), *n* (%) ^‡^	17 (81.0)	7 (77.8)	1.000	OR = 1.21 [0.18–8.22]
IFG or DM, *n* (%) ^‡^	12 (57.1)	6 (66.7)	0.704	OR = 0.67 [0.13–3.41]

Continuous variables are presented as median [minimum–maximum]. Categorical variables are presented as *n* (%). ^†^ Mann–Whitney U test; ^‡^ Fisher exact test. Bold *p*-values indicate statistical significance (*p* ≤ 0.05). Effect sizes: r = Z/√N for nonparametric comparisons; OR for categorical comparisons. NMF group included patients with no or mild fibrosis (F0–F1) and CSF group included those with clinically significant fibrosis (≥F2). BMI: body mass index; IFG: impaired fasting glucose; DM: diabetes mellitus; HOMA-IR: homeostatic model assessment for insulin resistance; CI: confidence interval.

**Table 2:** Laboratory Findings, Transient Elastography, and Non-Invasive Fibrosis Scores.

Variable	NMF Group (*n* = 21)	CSF Group (*n* = 9)	*p*	Effect Size [95% CI]
***Liver enzymes***				
AST (U/L) ^†^	23.0 [11.0–54.0]	32.0 [20.0–69.0]	0.048	r = 0.36 [0.05–0.63]
ALT (U/L) ^†^	31.0 [10.0–56.0]	35.0 [15.0–57.0]	0.634	r = 0.09 [0.01–0.45]
GGT (U/L) ^†^	41.0 [15.0–232.0]	62.0 [18.0–156.0]	0.365	r = 0.17 [0.01–0.51]
Elevated AST (>40 U/L), *n* (%) ^‡^	1 (4.8)	4 (44.4)	0.019	OR = 14.15 [1.11–825.63]
Elevated ALT (>33 U/L), *n* (%) ^‡^	10 (47.6)	5 (55.6)	0.999	OR = 0.73 [0.13–4.21]
Elevated GGT (>45 U/L), *n* (%) ^‡^	10 (47.6)	6 (66.7)	0.440	OR = 0.45 [0.08–2.25]
***Metabolic parameters***				
Fasting glucose (mg/dL) ^†^	103.0 [78.0–264.0]	104.0 [75.0–227.0]	0.946	r = 0.02 [0.01–0.44]
Fasting insulin (µU/L) †	18.0 [6.0–61.0]	24.0 [10.0–58.0]	0.353	r = 0.17 [0.01–0.51]
HOMA-IR ^†^	4.10 [1.25–25.40]	7.40 [1.96–14.40]	0.402	r = 0.16 [0.01–0.51]
HbA1c (%) ^†^	5.90 [5.00–10.00]	5.90 [5.00–10.00]	>0.999	r = 0.00 [0.01–0.45]
***Lipid profile***				
LDL (mg/dL) ^†^	128.0 [88.0–212.0]	92.0 [65.0–188.0]	0.141	r = 0.27 [0.01–0.68]
HDL (mg/dL) ^†^	51.0 [33.0–88.0]	48.0 [35.0–68.0]	0.541	r = 0.12 [0.01–0.45]
Triglycerides (mg/dL) ^†^	162.0 [52.0–405.0]	87.0 [72.0–242.0]	0.342	r = 0.18 [0.01–0.51]
***Other biochemistry***				
TSH (mIU/L) ^†^	2.09 [0.70–6.20]	2.07 [0.69–3.05]	0.651	r = 0.09 [0.01–0.44]
Albumin (g/dL) ^†^	4.50 [3.60–5.00]	4.33 [4.00–5.39]	0.682	r = 0.08 [0.01–0.42]
Ferritin (ng/mL) ^†^	81.0 [15.0–336.0]	43.0 [17.0–118.0]	0.096	r = 0.31 [0.03–0.59]
***Hematologic/Coagulation***				
Platelet count (×10^3^/µL) ^†^	257.0 [119.0–448.0]	126.0 [98.0–230.0]	<0.001	r = 0.67 [0.43–0.82]
MPV (fL) ^†^	8.80 [7.00–12.00]	9.40 [7.00–12.00]	0.388	r = 0.16 [0.01–0.50]
PT (sec) ^†^	11.60 [10.00–29.00]	12.20 [11.00–14.00]	0.153	r = 0.26 [0.02–0.58]
***Transient elastography***				
TE (kPa) ^†^	6.10 [4.80–13.60]	17.30 [5.60–53.70]	<0.001	r = 0.67 [0.39–0.82]
TE fibrosis stage (≥F3), *n* (%) ^‡^	1 (4.8)	8 (88.9)	**<0.001**	OR = 0.01 [0.01–0.07]
***Non-invasive fibrosis scores ^γ^***				
FIB-4 ^†^	1.05 [0.50–2.88]	2.51 [1.28–4.78]	<0.001	r = 0.67 [0.45–0.80]
FIB-4 ≥ 1.3, *n* (%) ^‡^	5 (23.8)	8 (88.9)	**0.002**	OR = 0.04 [0.01–0.26]
NFS ^†^	−1.239 [−3.468–0.409]	0.129 [−3.195–2.400]	0.006	r = 0.51 [0.15–0.76]
NFS ≥ −0.676, *n* (%) ^‡^	7 (33.3)	8 (88.9)	**0.014**	OR = 0.06 [0.02–0.44]
APRI ^†^	0.28 [0.11–0.82]	0.71 [0.28–1.18]	<0.001	r = 0.67 [0.42–0.82]
APRI ≥0.5, *n* (%) ^‡^	1 (4.8)	7 (77.8)	**<0.001**	OR = 0.01 [0.01–0.13]
BARD (score) ^†^	2 [0–4]	2 [2–4]	0.434	r = 0.14 [0.01–0.44]
BARD ≥ 2, *n* (%) ^‡^	15 (71.4)	9 (100.0)	0.141	OR: n.e.

Continuous variables are presented as median [minimum–maximum]. Categorical variables are presented as *n* (%). BARD is reported as median [minimum–maximum] as an ordinal variable. ^†^ Mann–Whitney U test; ^‡^ Fisher exact test. Bold *p*-values indicate statistical significance (*p* ≤ 0.05). Effect sizes: r = Z/√N for nonparametric comparisons; OR for categorical comparisons. NMF group included patients with no or mild fibrosis (F0–F1) and CSF group included those with clinically significant fibrosis (≥F2). AST: aspartate aminotransferase; ALT: alanine aminotransferase; GGT: gamma-glutamyl transferase; HOMA-IR: homeostatic model assessment for insulin resistance; TE: transient elastography; NFS: NAFLD fibrosis score; APRI: AST-to-platelet ratio index; CI: confidence interval. n.e. = not estimable. Zero cell ***^γ^***: All group differences that reached statistical significance held up under 10,000-iteration permutation testing; none of the initially significant findings were reversed.

**Table 3:** Individual Diagnostic Performance of Non-Invasive Tests for Clinically Significant Fibrosis (≥F2).

Test	AUROC	95% CI	Cutoff	Sens (%)	95% CI	Spec (%)	95% CI	PPV (%)	NPV (%)	+LR [95% CI]	−LR [95% CI]
TE (kPa)	0.93	[0.79–1.00]	≥10.70	88.9	[51.8–99.7]	90.5	[69.6–98.8]	80.0	95.0	9.33 [2.45–35.59]	0.12 [0.02–0.78]
FIB-4	0.93	[0.81–1.00]	≥1.28	100.0	[66.4–100.0]	76.2	[52.8–91.8]	64.3	100.0	4.20 [1.95–9.03]	0.00
NFS	0.83	[0.59–0.98]	≥−0.45	88.9	[51.8–99.7]	71.4	[47.8–88.7]	57.1	93.8	3.11 [1.52–6.36]	0.16 [0.02–1.01]
APRI	0.93	[0.80–1.00]	≥0.48	88.9	[51.8–99.7]	95.2	[76.2–99.9]	88.9	95.2	18.67 [2.72–128.18]	0.12 [0.02–0.74]
BARD	0.59	[0.39–0.78]	≥2	100.0	[66.4–100.0]	28.6	[11.3–52.2]	37.5	100.0	1.40 [1.07–1.83]	0.00
TE categorical ^a^	—	—	F ≥ 3	88.9	[51.8–99.7]	95.2	[76.2–99.9]	88.9	95.2	18.67 [2.72–128.18]	0.12 [0.02–0.74]

^a^ TE categorical classification based on device fibrosis stage ≥F3; AUROC not calculated. Sensitivity and specificity 95% CIs calculated by the Clopper-Pearson exact method. AUC 95% CIs calculated by 2000-iteration bootstrap (percentile method). PPV and NPV are based on sample prevalence (30.0%). +LR and −LR CIs calculated by the Simel method. −LR = 0.00 indicates 100% sensitivity with no missed cases. TE: transient elastography; NFS: NAFLD fibrosis score; APRI: AST-to-platelet ratio index; AUROC: area under the receiver operating characteristic curve; Sens: sensitivity; Spec: specificity; PPV: positive predictive value; NPV: negative predictive value; LR: likelihood ratio; CI: confidence interval.

**Table 4:** Classification Performance of Non-Invasive Indices According to Guideline Dual Cutoff Values.

Test	Category	*n*	No/Mild Fibrosis (F0–F1)	Clinically Significant Fibrosis (≥F2)	Adv. Rate	Missed	Gray Zone
FIB-4 (<1.30/>2.67)	Low	17	16	1	5.9%	1/9 (11.1%)	8/30 (26.7%)
	*Gray*	8	4	4	50.0%		
	High	5	1	4	80.0%		
NFS (<−1.455/>0.676)	Low	9	8	1	11.1%	1/9 (11.1%)	17/30 (56.7%)
	*Gray*	17	13	4	23.5%		
	High	4	0	4	100.0%		
APRI (<0.50/>1.00)	Low	22	20	2	9.1%	2/9 (22.2%)	6/30 (20.0%)
	*Gray*	6	1	5	83.3%		
	High	2	0	2	100.0%		
BARD (<2/≥2)	Low	6	6	0	0.0%	0/9 (0.0%)	—
	High	24	15	9	37.5%		
TE categorical (F0–2/F3–4)	Mild	21	20	1	4.8%	1/9 (11.1%)	—
	Advanced	9	1	8	88.9%		

FIB-4, NFS, and APRI cutoff values are based on EASL 2021 and AASLD 2023 guidelines [22,23]. BARD ≥2 is the original study cutoff. TE categorical classification is based on device fibrosis stage ≥F3. Missed: clinically significant fibrosis (≥F2) patients classified into the low/mild category. Gray zone: proportion of patients with indeterminate classification.

**Table 5:** Classification Matrix (Confusion Matrix).

Test	TP/9 (Sens)	FN/9 (Missed)	FP/21	TN/21 (Spec)	Accuracy
TE (kPa) ^a^	8 (88.9%)	1 (11.1%)	2	19 (90.5%)	90.0%
FIB-4 ^a^	9 (100.0%)	0 (0.0%)	5	16 (76.2%)	83.3%
NFS ^a^	8 (88.9%)	1 (11.1%)	6	15 (71.4%)	76.7%
APRI ^a^	7 (77.8%)	2 (22.2%)	1	20 (95.2%)	90.0%
BARD ^a^	9 (100.0%)	0 (0.0%)	15	6 (28.6%)	50.0%
HOMA-IR ^b^	7 (77.8%)	2 (22.2%)	17	4 (19.0%)	36.7%
TE categorical ^c^	8 (88.9%)	1 (11.1%)	1	20 (95.2%)	93.3%

^a^ Classified based on Youden index optimal cutoffs from Table 3 (TE ≥10.70 kPa; FIB-4 ≥ 1.28; NFS ≥ −0.45; APRI ≥ 0.48; BARD ≥ 2). ^b^ Classified based on clinical cutoff (HOMA-IR ≥2.5). ^c^ Classified based on device fibrosis stage ≥F3. TP: true positive; FN: false negative; FP: false positive; TN: true negative; Sens: sensitivity; Spec: specificity.

## Data Availability

The data presented in this study are available from the corresponding author upon reasonable request. The data are not publicly available because they contain information that could compromise participant privacy.

## Supplementary Materials

The following supporting information can be downloaded at: https://www.mdpi.com/article/10.3390/medicina62081527/s1, Table S1. Transient elastography and serum-based markers by individual histological fibrosis stage; Table S2. Diagnostic performance restricted to isolated stage F2 fibrosis.
