# Supplementary material for: Noninvasive Detection of Clinically Significant Fibrosis in Biopsy-Proven MASLD: A Retrospective Cross-Sectional Diagnostic Accuracy Study
Source: Medicina (Kaunas). 2026 Aug 8;62(8):1527. doi: 10.3390/medicina62081527 (PMC13515938; doi:10.3390/medicina62081527)
Supplement: Supplementary file 1 [file medicina-62-01527-s001.zip › EDT25192_Supplementary_Table_S1.pdf]

Table S1. Transient elastography and serum-based markers by individual histological fibrosis stage.

Variables	F0 (n=16)	F1 (n=5)	F2 (n=6)	F3 (n=3)
**TE (kPa)**	5.5 [4.8–13.6]	9.2 [5.6–10.9]	16.4 [5.6–35.8]	35.1 [34.0–53.7]
**FIB-4**	1.04 [0.50–2.88]	1.17 [0.58–1.85]	2.19 [1.28–4.78]	3.10 [1.70–3.18]
**NFS**	−1.53 [−3.47–0.41]	−0.65 [−1.25–0.16]	0.12 [−3.20–2.40]	1.33 [−0.17–1.37]
**APRI**	0.27 [0.11–0.82]	0.31 [0.18–0.47]	0.63 [0.28–1.18]	0.71 [0.56–0.87]
**BARD**	2 [0–4]	2 [1–3]	3 [2–4]	2 [2–2]
**AST (U/L)**	24.0 [11.0–39.0]	23.0 [19.0–54.0]	35.0 [20.0–69.0]	32.0 [23.0–44.0]
**ALT (U/L)**	29.5 [10.0–56.0]	38.0 [26.0–50.0]	32.5 [15.0–52.0]	39.0 [21.0–57.0]
**GGT (U/L)**	48.5 [15.0–232.0]	31.0 [19.0–59.0]	65.0 [18.0–156.0]	45.0 [27.0–47.0]
**Platelet (×10³/µL)**	264.5 [119.0–448.0]	209.0 [186.0–285.0]	138.5 [98.0–230.0]	112.0 [102.0–126.0]
**HOMA-IR**	3.75 [1.25–11.80]	10.60 [3.40–25.40]	7.70 [1.96–14.40]	6.40 [1.97–9.45]
**Age (years)**	60.0 [52.0–70.0]	59.0 [42.0–69.0]	56.5 [29.0–71.0]	51.0 [47.0–67.0]
**BMI (kg/m²)**	29.0 [24.0–40.0]	35.0 [30.0–38.0]	28.5 [20.0–35.0]	31.0 [26.0–40.0]
**Albumin (g/dL)**	4.5 [3.6–5.0]	4.6 [4.3–4.8]	4.4 [4.0–5.4]	4.3 [4.0–4.4]
**Patients above the study cutoff, n (%)**				
TE ≥10.70 kPa	1 (6.3)	1 (20.0)	5 (83.3)	3 (100.0)
FIB-4 ≥1.28	3 (18.8)	2 (40.0)	6 (100.0)	3 (100.0)
NFS ≥−0.45	4 (25.0)	2 (40.0)	5 (83.3)	3 (100.0)
APRI ≥0.476	1 (6.3)	0 (0.0)	5 (83.3)	3 (100.0)
BARD ≥2	11 (68.8)	4 (80.0)	6 (100.0)	3 (100.0)
TE stage ≥F3 (device)	1 (6.3)	0 (0.0)	5 (83.3)	3 (100.0)

Values are presented as median [minimum–maximum]. No patient had stage F4 fibrosis. The cutoffs listed in the lower panel are those derived by the Youden index in the main analysis (Table 3); the categorical transient elastography variable is the fibrosis stage recorded on the device report. Stage-wise comparisons were not tested because of the number of patients per stage. ALT: alanine aminotransferase; APRI: AST-to-platelet ratio index; AST: aspartate aminotransferase; BMI: body mass index; FIB-4: Fibrosis-4 index; GGT: gamma-glutamyl transferase; HOMA-IR: homeostatic model assessment for insulin resistance; NFS: NAFLD fibrosis score; TE: transient elastography.
