# Supplementary material for: Noninvasive Detection of Clinically Significant Fibrosis in Biopsy-Proven MASLD: A Retrospective Cross-Sectional Diagnostic Accuracy Study
Source: Medicina (Kaunas). 2026 Aug 8;62(8):1527. doi: 10.3390/medicina62081527 (PMC13515938; doi:10.3390/medicina62081527)
Supplement: Supplementary file 1 [file medicina-62-01527-s001.zip › EDT25192_Supplementary_Table_S2.pdf]

Table S2. Diagnostic performance restricted to isolated stage F2 fibrosis.

Tests	AUROC [95% CI]	Cutoff	Sensitivity (%)	Specificity (%)	F2 identified (n/6)
**TE (kPa)**	0.89 [0.69–1.00]	≥10.70	83.3	90.5	5
**FIB-4**	0.90 [0.76–1.00]	≥1.28	100.0	76.2	6
**NFS**	0.77 [0.47–0.98]	≥−0.45	83.3	71.4	5
**APRI**	0.90 [0.73–1.00]	≥0.476	83.3	95.2	5
**BARD**	0.67 [0.46–0.86]	≥2	100.0	28.6	6
**TE categorical**	—	Stage ≥F3	83.3	95.2	5

The three patients with stage F3 fibrosis were excluded, leaving stage F2 (n=6) compared with stages F0–F1 (n=21). Areas under the curve and their confidence intervals were obtained from 2000 bootstrap resamples by the percentile method. Cutoffs are those derived by the Youden index in the main analysis. The categorical transient elastography variable is the fibrosis stage recorded on the device report, for which no area under the curve was calculated. This analysis was performed in response to peer review and is reported as post hoc. APRI: AST-to-platelet ratio index; AUROC: area under the receiver operating characteristic curve; CI: confidence interval; FIB-4: Fibrosis-4 index; NFS: NAFLD fibrosis score; TE: transient elastography.
